# Potential Antioxidant and Anti-Inflammatory Properties of Polyphenolic Compounds from *Cirsium japonicum* Extract

**DOI:** 10.3390/ijms25020785

**Published:** 2024-01-08

**Authors:** Hun Hwan Kim, Se Hyo Jeong, Min Yeong Park, Pritam Bhagwan Bhosale, Abuyaseer Abusaliya, Hyun Wook Kim, Je Kyung Seong, Dong Il Kim, Sang Joon Lee, Kwang Il Park, Gon Sup Kim

**Affiliations:** 1 Research Institute of Life Science and College of Veterinary Medicine, Gyeongsang National University, Jinju 52828, Republic of Koreatpgy123@gmail.com (S.H.J.); lilie17@daum.net (M.Y.P.); shelake.pritam@gmail.com (P.B.B.); yaseerbiotech21@gmail.com (A.A.); kipark@gnu.ac.kr (K.I.P.); 2 Division of Animal Bioscience & Intergrated Biotechnology, Gyeongsang National University, Jinju 52725, Republic of Korea; 3 Laboratory of Developmental Biology and Genomics, BK21 PLUS Program for Creative Veterinary Science Research, Research Institute for Veterinary Science, College of Veterinary Medicine, Seoul National University, Seoul 08826, Republic of Korea; snumouse@snu.ac.kr; 4 Namhae Garlic Research Institute, 2465-8 Namhaedaero, Namhae 52430, Republic of Korea; kimdongi1@hanmail.net; 5 Gyeongnam Department of Environment Toxicology and Chemistry, Biological Resources Research Group, Korea Institute of Toxicology, 17 Jegok-gil, Jinju 52834, Republic of Korea; sjlee@kitox.re.kr

**Keywords:** HPLC-MS/MS, polyphenol compounds, antioxidant, anti-inflammation, *Cirsium japonicum*

## Abstract

*Cirsium japonicum *is a medicinal plant that has been used due to its beneficial properties. However, extensive information regarding its therapeutic potential is scarce in the scientific literature. The antioxidant and anti-inflammatory potential of polyphenols derived from the *Cirsium japonicum *extracts (CJE) was systematically analyzed. High-performance liquid chromatography (HPLC) with mass spectrometry (MS) was used to examine the compounds in CJE. A total of six peaks of polyphenol compounds were identified in the extract, and their MS data were also confirmed. These bioactive compounds were subjected to ultrafiltration with LC analysis to assess their potential for targeting cyclooxygenase-2 (COX2) and DPPH. The outcomes showed which primary compounds had the highest affinity for binding both COX2 and DPPH. This suggests that components that showed excellent binding ability to DPPH and COX2 can be considered significant active substances. Additionally, in vitro analysis of CJE was carried out in macrophage cells after inducing inflammation with lipopolysaccharide (LPS). As a result, it downregulated the expression of two critical pro-inflammatory cytokines, COX2 and inducible nitric oxide synthase (iNOS). In addition, we found a solid binding ability through the molecular docking analysis of the selected compounds with inflammatory mediators. In conclusion, we identified polyphenolic compounds in CJE extract and confirmed their potential antioxidant and anti-inflammatory effects. These results may provide primary data for the application of CJE in the food and pharmaceutical industries with further analysis.

## 1. Introduction

*Cirsium japonicum* is a wild perennial Asteraceae plant native to Northeast Asia. The leaves and roots have been widely used as medicine since ancient times, because they protect the liver, improve digestion and improve blood vessels and health [1,2]. Studies show that it lowers blood pressure and improves heart disease in hypertensive patients [3]. According to a recent study, *Cirsium japonicum* contains large amounts of polyphenolic compounds such as flavonoids and is considered a promising plant for its anti-inflammatory and antioxidant properties [4,5]. These phenolic compounds are secondary metabolites produced through phenylpropanoid metabolism in plants and have one or more hydroxyl substituents and a benzene ring. These phenolic compounds exist in various forms, from single phenol molecules to polyphenols [6]. As the side effects of synthetic drugs that inhibit oxidative and inflammatory reactions are revealed, antioxidants and anti-inflammatory agents based on natural compounds are emerging [2,7].

The increase in reactive oxygen species (ROS) causes cell and tissue damage, which induces an inflammatory response and results in a continuous effect, and oxidative stress and inflammatory mechanisms form a significant correlation [8,9]. The inflammatory response is an immune response to a stimulus. Lipopolysaccharide (LPS) is a substance that causes inflammation in macrophages and makes up the cell membrane of Gram-negative bacteria. Among them, when LPS induces inflammation, it binds to CD14, the host’s LPS-binding protein, and then activates pathways such as NF-κB and MAPK through Toll-like receptor 4. Inflammatory mediators nitric oxide (NO) and prostaglandins (PGE2) are activated, followed by inflammatory cytokines such as cyclooxygenase-2 (COX2) and inducible nitric oxide synthase (iNOS) [10,11,12]. Therefore, many inflammation experiments using LPS are conducted, and the anti-inflammatory and antioxidant effects of *Cirsium japonicum* are already known and used in many studies [13]. However, the specific polyphenol compounds that exhibit the main role of antioxidant and anti-inflammatory effects and the mechanism of each compound in terms of structure and binding are still unknown.

Molecular docking shows high accuracy when predicting the shape and affinity of the bond between the protein surface and drug through structural bonding [14]. This analytical bioinformatics modeling is used to predict the binding affinity of a ligand complex to its target receptor [15]. There are two main methods of molecular docking: one is to analyze the complementary surface area binding between a protein and a ligand, and the other is to show the actual bond in which the pairwise mutual energy of a ligand and a protein is calculated [16,17]. Therefore, an essential point in molecular docking analysis is that the scores shown in the modeled values do not necessarily have accurate binding strength, so a procedure must be used to confirm actual structural binding [18].

Therefore, this study focuses on the identification of polyphenols present in *Cirsium Japonicum *and the determination of those that play a leading role in its beneficial properties including antioxidant and anti-inflammatory activity. Our study method is differentiated from existing research methods. Additionally, it is challenging to comprehend the competitive interaction between chemicals, because standard substances verify the physiological activity of each. However, the chemicals in natural material extracts are subjected to a competitive reaction. As a result of our study approach, researchers can rapidly and precisely identify which compounds are most competitive. This approach will help effectively screen compounds present in extracts using active chemicals found in natural products and can be effectively applied in the early screening stages of drug development. This study aims to screen compounds with potential antioxidant and anti-inflammatory responses in *Cirsium japonicum* extract and then support the anti-inflammatory effect through additional in vitro and molecular docking data.

## 2. Results

### 2.1. Separation and Characterization of Polyphenol Compounds in Cirsium japonicum Extract

**Figure 1:**
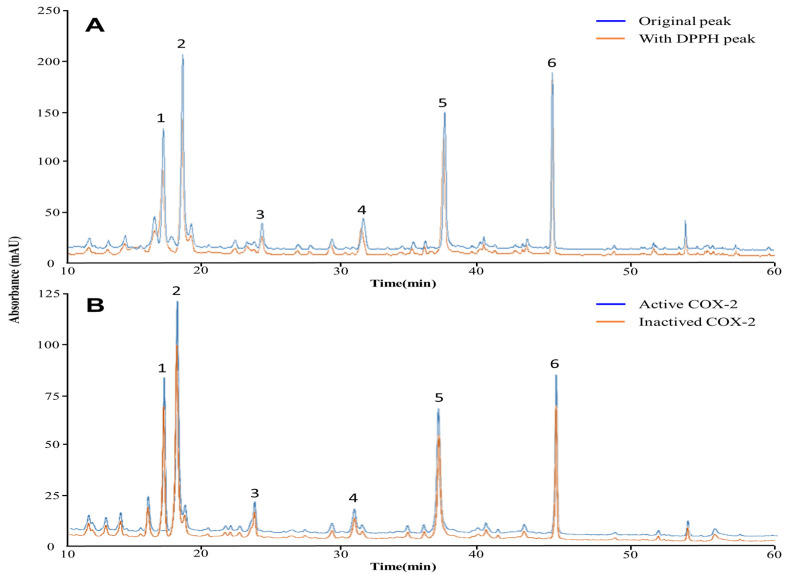
The HPLC chromatograms of the phenolic compounds in *Cirsium japonicum* extract. (**A**) The chromatogram at the beginning of the *Cirsium japonicum* extract is shown in blue, and the chromatogram following the reaction with the DPPH solution is shown in orange. (**B**) The chromatogram of the *Cirsium japonicum* extract reacted with activated COX2 is shown in blue, and the chromatogram following the reaction with the inactivated COX2 is shown in orange. The detected compounds at the 284 nm wavelength are syringin (1), chlorogenic acid (2), 3,5-Di-caffeoyl quinic acid (3), 3′-Hydroxycinnamic acid (4), 3,4-Di-caffeoylquinic acid (5), and oenin (6).

Qualitative analysis of compounds contained in *Cirsium japonicum* extract was performed by HPLC-MS/MS. UV–Vis spectra and HPLC retention time yielded a total of six major peaks (Figure 1). HPLC was used to identify six compounds contained in the extract at a wavelength of 284 nm. Table 1 shows the mass spectrometry identification of six compounds from published sources. There were six compounds, including syringin [19], chlorogenic acid [20], 3,5-Di-caffeoylquinic acid [21], 3′-Hydroxycinnamic acid [22], 3,4-Di-caffeoylquinic acid [23], and oenin [24].

**Figure 2:**
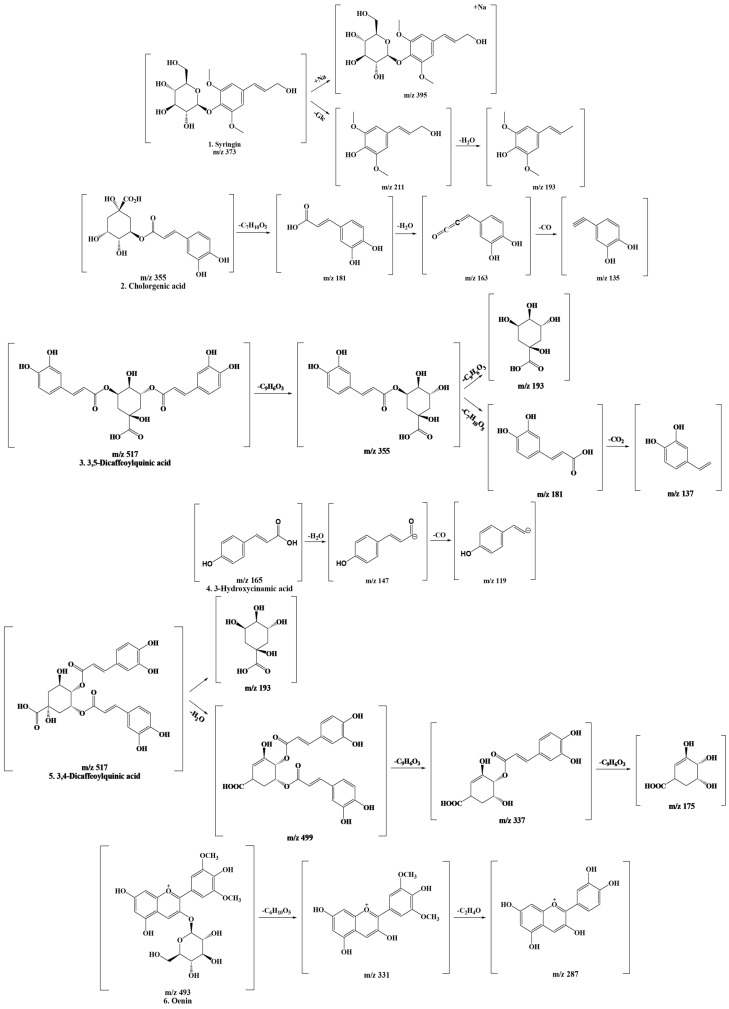
Fragmentation scheme of the polyphenolic compounds contained in *Cirsium japonicum* extract.

In this experiment, we focused on characterizing the detected compounds and analyzed data based on molecular ions and mass patterns obtained through LC-MS/MS data. Based on these results, Figure 2 shows the results of predicting the fragmentation of the compound. The fragmentation pattern served as the basis for the formation of compounds. The structural properties of the phenolic compounds identified in this extract were investigated.

### 2.2. Screening of Antioxidant Polyphenolic Compounds in Cirsium japonicum Extract

2,2-Diphenyl-1-picrylhdrazyl (DPPH) reagent is mainly used to analyze natural products’ radical scavenging or antioxidant effects. Antioxidants provide electrons to free radicals, and the color of the reagent changes from purple to yellow to evaluate its antioxidant capacity [25]. Screening for candidates with potential antioxidant effects among polyphenol compounds from *Cirsium japonicum* extract was conducted by combining DPPH and HPLC. HPLC peak area values were compared and screened before and after the polyphenol component and DPPH reaction.

Based on HPLC-MS/MS results obtained after reacting DPPH with *Cirsium japonicum* extract in Figure 1A, all selected polyphenol compounds in the extract showed changes in peak area values, contributing to a potential role in the antioxidant effect. In Table 2, the decrease in peak area after reaction demonstrates the antioxidant effect of the polyphenol compounds that reacted competitively with DPPH, and the difference in area values (%) indicates excellent radical scavenging ability. The polyphenols that specifically reacted highly with DPPH were chlorogenic acid (376.6), syringin (255.6), and 3,4-Di-caffeoylquinic acid (117.4) in Table 2. However, the relative peak area ratio difference was highest for syringin and 3,5-Di-caffeoylquinic acid at 12.22% and 12.18%, respectively, followed by chlorogenic acid at 11.12%. 3′-Hydroxycinnamic acid and oenin showed insignificant differences in the change in peak area value of binding with DPPH at 2.66% and 2.85%, respectively. This shows that syringin and 3,5-Di-caffeoylquinic acid combined with DPPH in a relatively more significant proportion, showing high antioxidant potential. On the other hand, 3′-Hydroxycinnamic acid and oenin showed slight differences before and after the reaction, showing a lower antioxidant response than other polyphenol compounds.

### 2.3. Screening of Anti-Inflammatory Polyphenolic Compounds in Cirsium japonicum Extract

Cyclooxygenase-2 (COX2) is formed from arachidonic acid and is subsequently involved in the formation of prostaglandins, which are an extension of the inflammatory response. In this process, anti-inflammatory effects occur through the inhibition of COX2, which is an enzyme that plays a major role in the inflammation response [26]. Therefore, compounds contained in any material can inhibit the progression of prostaglandins from arachidonic acid through a reaction with COX2.

The peaks before and after the COX2 reaction in Figure 1B show the extent to which six selected polyphenolic compounds from *Cirsium japonicum *extract bound to COX2. The binding of the inflammatory marker to each compound demonstrates its potential anti-inflammatory effects. The area value of the COX2-activated peak increased compared to the area value of the COX2-deactivated peak, which is the result of the polyphenol compounds in the *Cirsium japonicum* extract binding to COX2. In Table 3, the absolute response area values of polyphenol compounds and COX2 were highest in the order: chlorogenic acid (1954), syringin (1123.67), and 3,4-Di-caffeoylquinic acid (623). The relative area difference reaction rates were also high at 14.3% and 13.17% for chlorogenic acid and syringin, respectively.

### 2.4. Anti-Inflammatory Effects of Cirsium japonicum Extract (CJE)

Potential antioxidant and anti-inflammatory candidate polyphenol compounds obtained from *Cirsium japonicum* extract were identified. An in vitro experiment was conducted by administrating the extract on RAW264.7 macrophage cells after inflammation induced by LPS to confirm the anti-inflammatory effect of the extract.

#### 2.4.1. Effects of *Cirsium japonicum* Extract (CJE) on the Viability of RAW264.7 Cells

**Figure 3:**
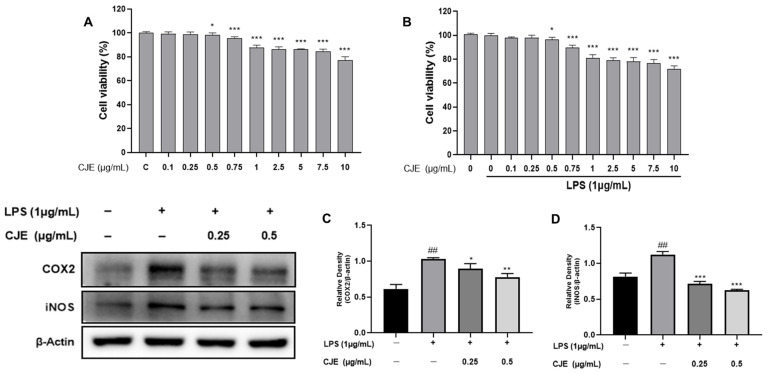
Cytotoxicity effects of CJE on RAW264.7 cells and the inhibition of inflammatory factors in LPS inflammation-induced RAW264.7 cells. RAW264.7 cells were pretreated without or with LPS (1 µg/mL) for 1 h at 37 °C. Following that, cells were treated with CJE (0, 0.1, 0.25, 0.5, 0.75, 1, 2.5 5, 7.5, and 10 µg/mL) for 24 h at 37 °C. (**A**) Cytotoxicity effects of CJE without LPS on RAW264.7 cells. (**B**) Cell viability in RAW264.7 cells treated with CJE and LPS. The RAW264.7 cells were treated with CJE (0, 0.25, and 0.5 μg/mL) at the indicated concentrations for 24 h. Cyclooxygenase-2 (COX2) and inducible nitric oxide (iNOS) levels were quantified. (**C**) The relative area of cyclooxygenase-2 (COX2). (**D**) The relative area of inducible nitric oxide (iNOS). Results from three independent experiments were expressed as mean ± standard error of the mean (SEM) compared with those of the control. ^##^ *p* < 0.01 vs. untreated group; * *p* < 0.05, ** *p* < 0.01, and *** *p* < 0.001 vs. LPS-treated group.

The cytotoxicity of the extract was confirmed by performing a 3-(3,4-dimethyl-thiazolyl-2)-2,5-diphenyl tetrazolium bromide (MTT) assay in RAW264.7 cells (Figure 3A,B). RAW 264.7 cells were treated with CJE at concentrations of 0, 0.1, 0.25, 0.5, 0.75, 1, 2.5, 5, 7.5, and 10 μg/mL for 24 h with or without 1 µg/mL of LPS. As a result, we found that the CJE was non-toxic at 0.25 and 0.5 µg/mL (Figure 3A). In Figure 3B, where inflammation was induced through LPS, the effect of the extract was confirmed at the same concentrations (0.25 and 0.5 µg/mL), and the concentrations were set and used in further experiments.

#### 2.4.2. *Cirsium japonicum* Extract (CJE) Inhibits COX2 and iNOS expression on LPS-Induced RAW264.7 Cells

Several studies have shown that the induction of inflammation through LPS results in the release of pro-inflammatory cytokines due to activation of the MAPK and NF-κB pathways [27]. In this process, pro-inflammatory cytokines iNOS and COX2 regulate inflammation [28,29]. Previous studies have shown that bioactive compounds are effective in anti-inflammatory and inhibit pro-inflammatory cytokines COX2 and iNOS in RAW264.7 cells treated with LPS [30]. Therefore, to determine the effects of CJE on COX2 and iNOS, we administered the extract at concentrations of 0.25 and 0.5 μg/mL after LPS treatment. The results showed that COX2 and iNOS were suppressed (Figure 3C,D), and in particular, a significant decrease in iNOS was observed in a dose-dependent manner.

### 2.5. Molecular Docking Analysis of Selected Polyphenol Compounds with NF-кB from Extracts

NF-κB is a protein complex involved in the production of inflammatory cytokines, and inappropriate regulation of NF-κB causes various inflammatory diseases [31]. There are various polyphenol compounds known to inhibit inflammation by targeting NF-κB [32]. Particularly in RAW 264.7 cells, LPS stimulation binds to Toll-like receptor 4 (TLR4), and downstream, under the influence of ROS, the NF-κB protein complex is upregulated, leading to the expression of COX2 and iNOS [33].

**Figure 4:**
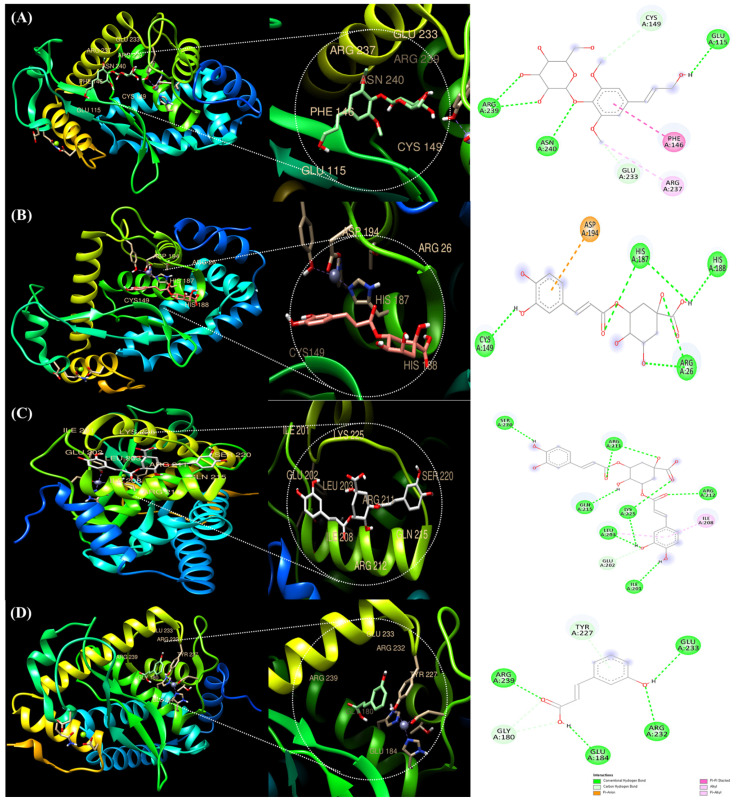
Molecular docking analysis of NF-κB and polyphenolic compounds in *Cirsium japonicum* extract. syringin (**A**), chlorogenic acid (**B**), 3,5-Di-caffeoylquinic acid (**C**), and 3′-Hydroxycinnamic acid (**D**) were all effectively bound to the 3D structure of NF-κB. Each color represents a direct bond type of interaction.

Therefore, molecular docking was performed with NF-κB using three polyphenol compounds (syringin, chlorogenic acid, and 3,5-Di-caffeoylquinic acid), which presented significant differences in area values among the six peaks previously identified in Figure 1. Additionally, 3′-Hydroxycinnamic acid was selected with a relatively low difference in area values (Figure 4). The binding of each protein and ligand to the complementary surface (Figure 4) and the mutual binding energy between the ligand and the protein are expressed as docking scores and are shown in Table 4.

Ligand–protein docking was performed using the UCSF Chimera program. Table 4 shows that the following active sites of syringin were bound to NF-κB *(*GLU233, ARG237, ARG239, ASN240, PHE146, CYS149, and GLU115). The molecular binding energy score with syringin was found to be −6.9 kcal/mol. The molecular docking chlorogenic acid to NF-κB revealed the following active sites (ASP194, HIS187, HIS188, ARG26, and CYS149) with a molecular binding energy score of −6.9 kcal/mol, the same as syringin. 3,5-Di-caffeoylquinic acid promoted NF-κB binding to the most abundant active sites (SER220, ARG211, ARG212, ILE208, LYS225, GLN215, LEU203, GLU202, and ILE201). The docking score also showed the highest molecular binding energy score of −7.5 kcal/mol.

The active sites of 3′-Hydroxycinnamic acid (ARG239, TYR227, GLU233, ARG232, GLU184, and GLY180) to NF-κB promoted the binding, and the binding energy score was the lowest at −5.5 kcal/mol.

## 3. Discussion

The structural properties of the phenolic compounds identified in this extract were investigated. First, syringin is a natural compound with the chemical structure of C_17_H_24_O_9_. Syringin, called Eleutheroside B, is a monosaccharide derivative and trans-sinapyl alcohol attached to the beta-D-glucopyranosyl residue at position 1 through a glycosidic bond [34,35]. Chlorogenic acid is a cinnamate ester with the chemical formula C_16_H_18_O_9_. Chlorogenic acid, also called 3-*O*-caffeoylquinic acid, is formed by the condensation reaction of the carboxy group of trans-caffeic acid and the 3′-hydroxy group of quinic acid and serves as an intermediate metabolite in lignin biosynthesis [36,37]. 3,5-Di-caffeoylquinic acid (isochlorogenic acid A) and 3,4-Di-caffeoylquinic acid (isochlorogenic acid B) both have the structural formula C_25_H_24_O_12_ [38]. In particular, 3,5-Di-caffeoylquinic acid is formed by condensation of the hydroxy group at positions 3 and 5 of quinic acid and the carboxyl group of trans-caffeic acid. 3,4-Di-caffeoylquinic acid is formed in the same reaction at positions 3 and 4 [39]. 3′-Hydroxycinnamic acid, also called M-coumaric acid, has the molecular formula of C_9_H_8_O_3_ [40]. This is a mono-hydroxycinnamic acid with a hydroxy substituent located at C3 of the phenyl ring [41]. Lastly, oenin has the molecular formula of C_23_H_25_O_12_^+^, the form of malvidin 3-glucoside cation with a sugar attached to the 3-position of malvidin [42].

In this study, the change in relative peak area before and after the reaction was conducted based on the existing literature, including the hypothesis that each peak indicates the reactivity of the compound and that this reaction indicates a potential antioxidant effect [43,44]. In these results, syringin showed the relatively largest difference in area values. 3,5-Di-caffeoylquinic acid also showed high binding activity. A recent study showed that the candidate ingredient, syringin, lowered oxidative stress in ischemia/reperfusion (I/R) rats, and 3,5-Di-caffeoylquinic acid shows antioxidant activity through radical scavenging and ferric reducing antioxidant power (FRAP) activity [45,46].

This study was conducted based on the existing comparative analysis method that enables the efficient discovery of ligands for specific peaks in the mixed compounds through UF-LC/MS after combining the COX2 enzyme and extract and performing rapid and accurate screening [47,48]. What is noteworthy is that 3,5-Di-caffeoylquinic acid showed a low response value of 251, but the response rate was high at 11.67%. In some studies, chlorogenic acid inhibits downstream inflammatory markers such as interleukin six among potential candidate polyphenolic components nuclear translocation of NF-κB [49]. In the case of syringin, it shows anti-inflammatory effects by suppressing the production of nitric oxide (NO), prostaglandin E2 (PGE2), and tumor necrosis factor-α (TNF-α) and stimulating the release of transforming growth factor-β (TGF-β) [30]. 3,5-Di-caffeoylquinic acid also showed anti-inflammatory effects by inhibiting the upregulation of iNOS, COX2, and TNF-α [46].

**Figure 5:**
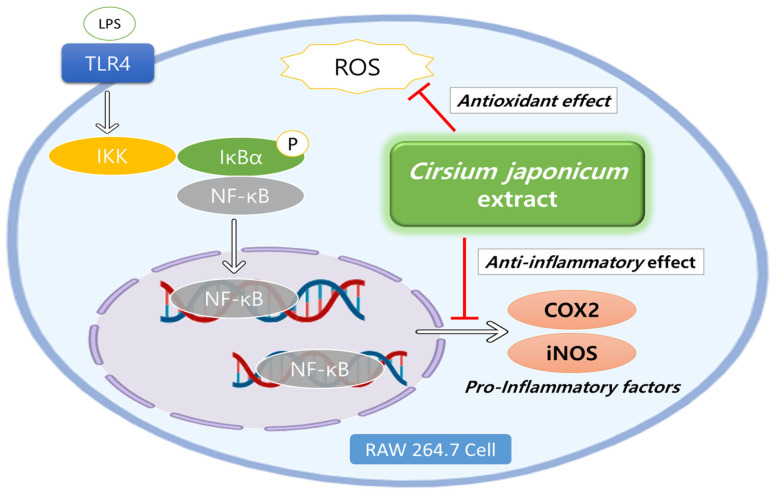
Schematic illustration of antioxidant and anti-inflammatory effects of *Cirsium japonicum* extract in RAW 264.7 Cells. This image was created by BioRender.

In this study, to confirm the anti-inflammatory effect of the extract itself, RAW 264.7 cells in which inflammation was induced with LPS were treated in a dose-dependent manner. Two anti-inflammatory markers, COX2 and iNOS, were significantly decreased, and NF-κB was selected as a docking candidate in the subsequent molecular docking through the following mechanism (Figure 5).

Through molecular docking of four selected polyphenolic compounds, the docking score of 3,5-Di-caffeoylquinic acid was relatively higher than those of syringin and chlorogenic acid, and the score of 3′-Hydroxycinnamic acid was the lowest. Therefore, in terms of structural binding, all four compounds showed potential anti-inflammatory effects, but among them, 3,5-Di-caffeoylquinic acid had the highest, and 3′-Hydroxycinnamic acid had the lowest. It is essential to determine the anti-inflammatory effect of polyphenolic compounds regarding structural binding through docking scores. Our result confirmed the scores by combining polyphenol compounds selected from CJE with inflammation-related proteins. These results suggest that the polyphenolic compounds in the extract have anti-inflammatory effects, not only in terms of potential antioxidant and anti-inflammatory activity, but also in terms of ligand–protein structure affinity. Furthermore, predictable docking results and in vitro results of the extract further support its potential anti-inflammatory effect. Thus, this extract could be proven as an effective drug against inflammatory diseases.

## 4. Materials and Methods

### 4.1. Plant Materials

Sancheong, Jirisan Mt, Gyeongsangnam-do, Korea, is the native habitat of *Cirsium japonicum, *and the plant was provided through the Animal Bio Resources Bank (Nationally Designated Research Materials Bank). The leaves and stems of the provided plants were rinsed with water, chopped and dried in a drying oven at 56 °C for 72 h. Afterwards, they were placed in sealed polyethylene bags containing a silica gel and stored at −20 °C until use.

### 4.2. Reagents, Chemicals, and Standards

The chemicals, COX2 enzymes and 2,2-Diphenyl-1-picrylhydrazyl (DPPH) reagent, were acquired from Sigma-Aldrich Corp. in St. Louis, MO, USA (cat no. 1898-66-4). A centrifugal ultrafiltration filter YM-30 (cat no. 42410) from Millipore Co., Ltd., Burlington, Middlesex County, MA, USA. with a 30 kDa capacity was bought. All other chemicals and solvents were of analytical grade and were obtained from Duksan Pure Chemical Co., Ltd. (Dongdaemun-gu, Seoul, Republic of Korea). Fetal bovine serum (FBS), phosphate-buffered saline (PBS), Dulbecco’s modified Eagle’s medium (DMEM), and penicillin/streptomycin (P/S) antibiotics were acquired from Gibco (BRL Life Technologies, Grand Island, NY, USA). COX2 (cat. no. 12282S), iNOS (cat. no. 13120S), and β-actin (cat. no. 3700S) antibodies were purchased from Cell Signaling Technology (Danvers, MA, USA). Secondary antibodies against rabbit and mouse were purchased from Bethyl Laboratories, Inc. (Montgomery, AL, USA) using horseradish peroxidase (HRP) conjugation.

### 4.3. Extraction Process of Cirsium japonicum and Purification of Polyphenol Components

Polyphenols were isolated from the plant using a modified technique [50]. First, 700 g of finely chopped *Cirsium japonicum* was used to extract 10 L of 70% ethanol for 4 days at room temperature (RT). The extract was filtered for impurities through filter paper (Whatman Qualitative No. 6). Afterwards, the extract was concentrated at 45 °C using a rotary evaporator (N-1110, Eyela, Tokyo, Japan), until it reached 500 mL. The concentrated extract was washed three times using 500 mL of hexane to remove fatty particles. Then, the remaining filtrate was extracted three times using 250 mL of ethyl acetate. The residue was first dehydrated with MgSO_4_ to remove the moisture and then eluted using a silica gel solvent (40 cm × 2.5 cm) and ethyl acetate. A mixed polyphenol powder was created by condensing the extract at a reduced pressure, and it was then kept at −70 °C. (48.2 g and 6.88% of the raw material).

### 4.4. HPLC and LC-MS/MS

For compound identification, 10 μL of the extract powder diluted in 70% ethanol to a concentration of 1000 μg/mL was used as a sample. HPLC and LC-MS/MS were performed on a 1260 series HPLC system (Agilent Technologies, Inc., Santa Clara, CA, USA) and Ultra Quadrupole Time of flight LC-MS/MS System (X500R) operated in the positive ion mode with a voltage set at −4.5 kV. The solvents used were distilled water (DW, solvent A) and acetonitrile containing 0.1% formic acid (solvent B), a gradient system was used at a flow rate of 0.5 mL/min for analysis, and a Prontosil C18 column (length: 250 mm, inner diameter: 4.6 mm, particle size: 5 µm; Phenomenex Co., Ltd., Torrance, CA, USA, Biochoff Chromatography) was used. The solvent B conditions used in the mobile phases were at 10–15% for 0–10 min, at 20% for 20–30 min, at 40% for 30–40 min, at 70% for 40–50 min, and at 95% for 50–60 min. The analysis was performed at a wavelength of 284 nm and a temperature of 35 °C.

### 4.5. DPPH-Binding HPLC Analysis for Measuring Main Antioxidant Activity of Polyphenolic Compounds

The antioxidant potential of compounds contained in *Cirsium japonicum* extract was determined using a modified technique [51]. A 1:1 (*v*:*v*) mixture of the polyphenolic compound (5000 μg/mL) in 70% ethanol and 0.2 mg/mL DPPH reagent was added, and the mixture was allowed to react at room temperature for 15 min. Before HPLC analysis of the combination, a filter with a pore size of 0.45 μm was employed for filtration, and methanol was utilized as a control in place of the DPPH reagent. The polyphenol compounds that reacted with DPPH could be identified by comparing the chromatographic peak area that underwent the DPPH reaction. After this, the main antioxidant polyphenolic compounds of *Cirsium japonicum *were identified.

### 4.6. Identification of the Reaction between Polyphenol Compounds and COX2 through UF-HPLC

A modified technology was used to evaluate the anti-inflammatory potential of compounds through binding to COX2 [52]. First, 100 μL of the extract diluted to 2000 μg/mL (containing 70% ethanol) and 20 μL of COX2 (2U) reacted in a water bath at 37 °C for 30 min. The control group reacted with inactivated COX2 through boiling water, and the experimental group reacted with non-inactivated COX2. The mixed solution was centrifuged at 10,000 rpm for 10 min at room temperature using 30 kd cut-off ultrafiltration (YM-30). After centrifugation, the solution that did not pass through the filter was washed three times with 200 μL NE buffer (pH 7.9, 25 °C) to remove unbound compounds. The solution remaining at the top after washing was a compound that bound to COX2 and did not pass through the filter, and the solution that passed through the filter was a compound that did not bind to COX2. Compounds remaining in the upper layer by binding to COX-2 were dissolved in 80% ACN for 10 min and extracted by centrifugation at 10,000 rpm for 10 min (repeated three times). The filtrate below was then analyzed via HPLC.

### 4.7. Measurement of Anti-Inflammatory Effects

#### 4.7.1. Cell Culture and Viability Assay

The RAW 264.7 cell line, a mouse macrophage, was acquired from the American Type Culture Collection (ATCC), cultured in complete DMEM with 10% FBS and supplemented with 100 U/mL penicillin and 100 μg/mL streptomycin (P/S). The cells were incubated at 37 °C in a humidified atmosphere with 5% CO_2_. RAW264.7 cells were seeded at a density of 1 × 10^4^ cells per well in 96-well plates for 12 h. Following this, the cells were exposed to *Cirsium japonicum* extract for 24 h at doses of 0, 0.1, 0.25, 0.5, 0.75, 1, 2.5, 5, 7.5, and 10 µg/mL, either with or without 1 µg/mL of LPS (Sigma-Aldrich, Merck KGaA, Burlington, MA, USA). Cells were incubated at 37 °C for 4 h after being treated with 10 µL of MTT solution (5 mg/mL, dissolved in PBS) in each well. Insoluble formazan crystals were dissolved in DMSO. Each sample was analyzed in triplicate, and absorbance (OD) values were read at 450 nm for each well using a microplate reader (BioTek, Winooski, VT, USA). All experiments were performed in triplicates.

#### 4.7.2. Western Blot Analysis

RAW264.7 cells were seeded into 60 mm plates at a density of 1 × 10^6^ cells per well and treated with 0.25 and 0.5 μg/mL *Cirsium japonicum *extract with or without 1 μg/mL of LPS (Sigma-Aldrich, Merck KGaA) for 24 h. Then, the cells were lysed using RIPA buffer (iNtRON Biotechnology Gyeonggi, Korea, 50 mM Tris-HCl (pH 8.0), 0.5% sodium deoxycholate, 1 mM EDTA, 150 mM NaCl, 0.1% SDS, and 1% NP-40) with a protease inhibitor cocktail and phosphatase inhibitor (Thermo Fisher Scientific in Waltham, MA, USA). Protein concentrations were determined using a bicinchoninic acid (BCA) protein assay kit (Thermo Fisher Scientific, Waltham, MA, USA). Equal amounts of 10 μg of the protein were separated using 10% Sodium Dodecyl Sulfate–PolyAcrylamide Gel Electrophoresis (SDS-PAGE). Using a semi-dry conveying system (Atto Corp., Tokyo, Japan), polyacrylamide gels were first poured, and then, they were transferred to polyvinylidene fluoride (PVDF) membranes. The membranes were then blocked for 2 h at room temperature using EzBlockChemi (ATTO Blotting System, Tokyo, Japan). In a subsequent step, membranes were treated with a 1:1000 diluted primary antibody overnight at 4 °C. The membrane reacted overnight was washed 5 times for 15 min at room temperature with TBS-T (Tween 20, pH 7.4) and then responded with anti-rabbit and anti-mouse antibodies diluted at 1:5000 for 3 h at room temperature. After completing the reaction, the membrane was washed 10 times for 2 h with TBS-T, then captured with a ChemiDoc imaging system (Version 6.0, Bio-Rad Laboratories, Inc., Hercules, CA, USA) with chemiluminescence (ECL) buffer (Bio-Rad, Hercules, CA, USA) and confirmed using the Image Lab 4.1 (Bio-Rad) application. Captured pictures were quantified using the Image J software (https://imagej.net/ij/, U. S. National Institutes of Health, Bethesda, MD, USA), and β-actin protein was used as a loading control. All experiments were performed in triplicates.

### 4.8. Molecular Docking Analysis

The protein structure from PDB was retrieved to do a molecular docking analysis (https://www.rcsb.org/, accessed on 20 August 2023) using the search ID 4Q3J (NF-κB). From PubChem (https://pubchem.ncbi.nlm.nih.gov, accessed on 10 May 2023), the 3D compound structures of syringin (Compound CID: 5316860), chlorogenic acid (Compound CID: 1794427), 3,5-Di-caffeoylquinic acid (Compound CID: 6474310), and 3′-Hydroxycinnamic acid (Compound CID: 637541) were downloaded. Docking analysis was performed using UCSF Chimera and AutoDock Vina with the default settings. PyMOL and Discovery Studio were used to display the docking results (DeLano, 2002) [51]. The affinity of the binding was calculated from the total intermolecular energy and the predicted free energy binding. All experiments were performed in triplicates and at a root-mean-square deviation (RSMD) of ≤2 Å.

### 4.9. Statistical Analysis

The mean and SEM were used to express the data. The data were analyzed using GraphPad Prism (version 9.3.1; GraphPad Software, Inc., Boston, MA, USA) software. Statistical analysis was carried out using SPSS version 12.0 (SPSS Inc., Chicago, IL, USA). They were examined using one-way factorial analysis of variance (ANOVA) to determine whether they differed significantly. The results of Dunnett’s multiple comparison tests were then evaluated, with a *p*-value of <0.05 being considered statistically significant (^#^ *p* < 0.05, ^##^ *p* < 0.01, ^###^ *p* < 0.001 vs. untreated, positive control group; and * *p* < 0.05, ** *p* < 0.01, *** *p* < 0.001 vs. LPS-treated, negative control group).

## 5. Conclusions

In this study, compounds with the potential to have significant antioxidant activity were discovered using a combined investigation of DPPH and HPLC on the polyphenols in CJE. Following the antioxidant analysis, the anti-inflammatory effects between the polyphenolic compounds were confirmed through an anti-inflammatory analysis using HPLC and a combination of COX2 and CJE. RAW 264.7 cells were treated with CJE and inflammation was induced through LPS, while COX2 and iNOS were confirmed to be downregulated. Additionally, it was established that the selected compounds of CJE had significant binding scores in structural binding by molecular docking with NF-κB, a representative inflammation-related receptor. We evaluated the antioxidant and anti-inflammatory properties of the ingredients in CJE to identify those with the most potential. These results suggest the potential use of the components contained in CJE in the pharmaceutical industry, while introducing innovative methods for other natural products. However, a small amount of compounds may be lost during impurities removal in the extraction process, thereby reducing the yield.

## Figures and Tables

**Table 1:** HPLC-MS/MS data for phenolic compounds of *Cirsium japonicum* extract.

Peak No.	Rt (min)	Formula	Compound	UV_max_	[M+H]^+^	MS/MS
1	15.98	C_17_H_24_O_9_	Syringin	268, 210	373	395 (C_17_H_24_O_9_Na) [M+H+Na]^+^ 211 (C_11_H_14_O_4_) [M+H-Glc]^+^ 193 (C_11_H_12_O_3_) [M+H-Glc-H_2_O]^+^
2	16.93	C_16_H_18_O_9_	Chlorogenic acid	325, 250	355	181 (C_9_H_8_O_4_) [M+H-C_7_H_10_O_5_]^+^ 163 (C_9_H_6_O_3_) [M+H-C_7_H_10_O_5_-H_2_O]^+^ 135 (C_8_H_6_O_2_) [M+H-C_7_H_12_O_6_-CO]^+^
3	22.25	C_25_H_24_O_12_	3,5-Di-caffeoylquinic acid	330, 290	517	355 (C_16_H_18_O_9_) [M+H-C_9_H_6_O_3_]^+^ 193 (C_7_H_12_O_6_) [M+H-C_9_H_6_O_3_-C_9_H_6_O_3_]^+^ 181 (C_9_H_8_O_4_) [M+H-C_9_H_6_O_3_-C_7_H_10_O_5_]^+^ 137 (C_8_H_8_O_2_) [M+H-C_16_H_16_O_8_-O]^+^
4	29.13	C_9_H_8_O_3_	3′-Hydroxycinnamic acid	320, 270	165	147 (C_9_H_6_O_2_) [M+H-H_2_O]^+^ 119 (C_8_H_6_O) [M+H-H_2_O-CO]^+^
5	34.99	C_25_H_24_O_12_	3,4-Di-caffeoylquinic acid	325, 290	517	499 (C_25_H_22_O_11_) [M+H-H_2_O]^+^ 337 (C_16_H_16_O_8_) [M+H-H_2_O-C_9_H_6_O_3_]^+^ 193 (C_7_H_12_O_6_) [M+H-C_18_H_12_O_6_]^+^ 175 (C_7_H_10_O_5_) [M+H-C_9_H_8_O_4_-C_9_H_6_O_3_]^+^
6	42.98	C_23_H_25_O_12_^+^	Oenin	515, 280	493	331 (C_17_H_15_O_7_^+^) [M+H-C_6_H_10_O_5_]^+^ 287 (C_15_H_11_O_6_^+^) [M+H-C_6_H_10_O_5_-C_2_H_4_O]^+^

Rt: retention time.

**Table 2:** Screening of antioxidant potential of *Cirsium japonicum* extract polyphenolic compounds.

Peak No.	Compound	Initial Area (Composition %)	Area after DPPH Reaction	Reactive Percentage (%)
1	Syringin	2091.50 ± 8.25 ^bD^ (18.95 ± 0.08)	1835.87 ± 6.53 ^aD^	12.22 ± 0.66 ^C^
2	Chlorogenic acid	3385.63 ± 12.21 ^bF^ (30.97 ± 0.11)	3009.03 ± 8.76 ^aF^	11.12 ± 0.36 ^C^
3	3,5-Di-caffeoylquinic acid	541.03 ± 1.44 ^bA^ (4.95 ± 0.01)	475.13 ± 4.15 ^aA^	12.18 ± 1.00 ^C^
4	3′-Hydroxycinnamic acid	703.63 ± 4.23 ^bB^ (6.44 ± 0.04)	684.90 ± 6.56 ^aB^	2.66 ± 1.51 ^A^
5	3,4-Di-caffeoylquinic acid	2438.50 ± 3.54 ^bE^ (22.31 ± 0.03)	2321.10 ± 6.85 ^aE^	4.81 ± 0.36 ^B^
6	Oenin	1790.33 ± 3.36 ^bC^ (16.38 ± 0.03)	1739.27 ± 4.80 ^aC^	2.85 ± 0.43 ^A^

All values are mean ± SD (n = 3). Means with different superscripts in the same column ^(A–F)^ and the same compound ^(a,b)^ are significantly different at *p* < 0.05 by Duncan’s multiple range tests. Composition %: the percentage composition of the individual compounds in the extract.

**Table 3:** Screening of anti-inflammatory potential of *Cirsium japonicum* extract polyphenolic compounds.

Peak No.	Compound	With an Active COX2 Area	With an Inactive COX2 Area	Area Reacted with COX2 (%)
1	Syringin	8532 ± 11.53 ^bD^	7408.33 ± 9.29 ^aD^	13.17 ± 0.22 ^CD^
2	Chlorogenic acid	13,664.67 ± 18.72 ^bF^	11,710.67 ± 17.21 ^aF^	14.30 ± 0.21 ^D^
3	3,5-Di-caffeoylquinic acid	2150 ± 16.82 ^bB^	1899 ± 13.53 ^aB^	11.67 ± 1.31 ^C^
4	3′-Hydroxycinnamic acid	1637.67 ± 21.73 ^bA^	1581.33 ± 14.05 ^aA^	3.43 ± 1.61 ^A^
5	3,4-Di-caffeoylquinic acid	9387.33 ± 18.90 ^bE^	8764.33 ± 11.50 ^aE^	6.64 ± 0.08 ^B^
6	Oenin	6976.67 ± 25.89 ^bC^	6490.33 ± 11.02 ^aC^	6.97 ± 0.48 ^B^

All values are mean ± SD (n = 3). Means with different superscripts in the same column ^(A–F)^ and the same compound ^(a,b)^ are significantly different at *p* < 0.05 by Duncan’s multiple range tests.

**Table 4:** Molecular docking of syringin, chlorogenic acid, 3,5-Di-caffeoylquinic acid, and 3′-Hydroxycinnamic acid with NF-κB protein complex and their binding energies.

Binding ligand	Amino Acid Residue that Interacts	Docking Score
Syringin	GLU233, ARG237, ARG239, ASN240, PHE146, CYS149, and GLU115	−6.9 kcal/mol
Chlorogenic acid	ASP194, HIS187, HIS188, ARG26, and CYS149	−6.9 kcal/mol
3,5-Di-caffeoylquinic acid	SER220, ARG211, ARG212, ILE208, LYS225, GLN215, LEU203, GLU202, and ILE201	−7.5 kcal/mol
3′-Hydroxycinnamic acid	ARG239, TYR227, GLU233, ARG232, GLU184, and GLY180	−5.5 kcal/mol

## Data Availability

The data used to support the findings of this study are available upon request from the corresponding author.
